# Prediction of chaperonin GroE substrates using small structural patterns of proteins

**DOI:** 10.1002/2211-5463.13590

**Published:** 2023-03-14

**Authors:** Shintaro Minami, Tatsuya Niwa, Eri Uemura, Ryotaro Koike, Hideki Taguchi, Motonori Ota

**Affiliations:** ^1^ Graduate School of Informatics Nagoya University Japan; ^2^ Cell Biology Center, Institute of Innovative Research Tokyo Institute of Technology Yokohama Japan; ^3^ Institute for Glyco‐core Research Nagoya University Japan

**Keywords:** chaperone, GroE, hydrophobic pattern, protein structural comparison, protein structure classification, protein super‐secondary structure

## Abstract

Molecular chaperones are indispensable proteins that assist the folding of aggregation‐prone proteins into their functional native states, thereby maintaining organized cellular systems. Two of the best‐characterized chaperones are the *Escherichia coli* chaperonins GroEL and GroES (GroE), for which *in vivo* obligate substrates have been identified by proteome‐wide experiments. These substrates comprise various proteins but exhibit remarkable structural features. They include a number of α/β proteins, particularly those adopting the TIM β/α barrel fold. This observation led us to speculate that GroE obligate substrates share a structural motif. Based on this hypothesis, we exhaustively compared substrate structures with the MICAN alignment tool, which detects common structural patterns while ignoring the connectivity or orientation of secondary structural elements. We selected four (or five) substructures with hydrophobic indices that were mostly included in substrates and excluded in others, and developed a GroE obligate substrate discriminator. The substructures are structurally similar and superimposable on the 2‐layer 2α4β sandwich, the most popular protein substructure, implying that targeting this structural pattern is a useful strategy for GroE to assist numerous proteins. Seventeen false positives predicted by our methods were experimentally examined using GroE‐depleted cells, and 9 proteins were confirmed to be novel GroE obligate substrates. Together, these results demonstrate the utility of our common substructure hypothesis and prediction method.

## Graphical abstract

Chaperonin GroE prefers proteins adopting α/β class structures as substrates. We assumed the common features in substrates by focusing on the local structures most exclusively included in substrates, and developed a GroE substrate predictor. The selected substructures are projectable onto a 2‐layer 2α4β sandwich, the most ubiquitous substructure in proteins, which explains the versatile role of GroE in folding.

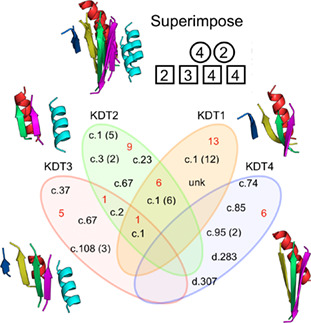

**Abbreviations**
2α4β: 2‐layer 2α4β sandwich
KD: Kyte‐Doolittle
KDT: Kyte‐Doolittle template
MCC: Matthew's correlation coefficient
NIP: normalized inner product
PDB: the Protein Data Bank
Roc plot: receiver‐operating characteristic plot
RR: rewiring and reverse
RW: rewiring
SCCS: SCOP concise classification string
SCIP: Solving Constraint Integer Programs
SCOP: structure classification of proteins
SQ: sequential
ΔSol: Δsolubility

Most proteins composed of structural domains are believed to fold into their unique structures encoded by their amino acid sequences *in vitro* [1]. By contrast, *in vivo* spontaneous protein folding is considered to be rather difficult within the crowded cellular environment [2]. Chaperones are an essential molecular system to prevent newly synthesized proteins from misfolding and aggregation, and they assist nascent protein folding to generate their functional native states [3, 4, 5]. Indeed, among more than 3000 *Escherichia coli* proteins synthesized by reconstituted cell‐free translation, a quarter was aggregation‐prone under chaperone‐free conditions [6]. By contrast, most of them were soluble in the presence of chaperones [7]. Protein aggregation could cause severely detrimental effects in living cells [8], and thus chaperones are absolutely necessary and found in all kingdoms of life. To understand the harmonized system of proteins in the cellular environment, uncovering the roles of chaperones is indispensable.

One of the best‐studied chaperones is the chaperonin GroEL/GroES (GroE) from *E. coli* [9, 10, 11, 12], composed of two stacked heptameric rings (GroEL) and a hatch‐like cofactor (GroES) [13]. GroEL has regions that bind non‐native states of proteins [14, 15, 16]. *In vitro*, purified GroEL can interact with about half of the soluble *E. coli* proteins upon dilution from denaturant [17], indicating that GroEL has an ability to bind a large fraction of non‐native states of proteins. The binding of GroEL to the proteins is a prerequisite for subsequent folding but not always sufficient. It has been known that the requirement of the chaperonin for the *in vitro* folding depends on the proteins [12]. For some proteins, folding proceeds with only GroEL and ATP. Whereas, a full set of the chaperonin system, GroEL, GroES, and ATP, is required for the productive folding of stringent substrates [18, 19, 20, 21]. Detailed analysis of certain stringent proteins revealed that even a small change in amino acid sequences could convert the GroE dependency [20, 22]. Although some features such as a local energetic frustration [22] or relative contact order [23] have been proposed to explain these results, the entire picture of the GroE dependency still remains obscure.

In addition to the detailed analysis of substrates *in vitro*, *in vivo* analyses have provided clues to clarify how GroE recognizes and assists them in *E. coli* cells. A proteome‐wide survey for a comprehensive list of GroE substrates has been conducted by mass spectrometry [21, 24]. Kerner *et al*. [21] have identified ~ 250 substrates that interact with GroE in cells, and categorized them into three classes based on their enrichment in the GroE complex: class I substrates as spontaneous folders, class II as partial GroE‐dependent substrates, and class III as the potential obligate GroE substrates. A further systematic survey of the class III substrates using a conditional GroE expression strain identified ~ 60 *in vivo* obligate GroE substrates, called class IV substrates [25]. In the analysis, the class IV substrates were defined as the proteins that form aggregates or the ones that are degraded under the GroE‐depleted conditions [25]. The evaluation method for the class IV substrates was further applied to the protein group extracted from data of the comprehensive analysis using the reconstituted cell‐free system and identified more than 20 class IV substrates [26]. The class IV GroE substrates are aggregation‐prone and have moderate MWs (< 69 kDa) and include proteins with diverse functions.

As mentioned above, GroE has highly promiscuous substrate recognition, binds a range of unfolded or partially folded proteins, and has some preference for the *in vitro* and *in vivo* substrates. Such substrate recognition would be crucially different from the common protein–protein recognition based on the complementarity of the molecular surfaces of folded structures [27, 28, 29].

A structural bioinformatic analysis of the GroE substrates revealed that GroE binds to various proteins with distinct folds but has a structural preference. SCOP (structure classification of proteins) classifies protein structural domains according to structural similarity and assigns SCOP concise classification string (SCCS) [30]. Interestingly, class III contains a number of proteins adopting the TIM β/α barrel fold [21] (SCCS: c.1). In addition, thiolase‐like (c.95), PLP‐dependent transferase‐like (c.67), and FAD/NAD(P)‐binding domain (c.3) folds are abundant in class IV [25, 26]. Roughly speaking, GroE prefers proteins whose structures consist of alternating α helices and β strands (α/β proteins) and whose structures with segregated α helices and β strands (α + β proteins), as compared with the ones whose structures involve only α helices (all α proteins) and β sheets (all β proteins). However, S‐adenosyl‐l‐methionine‐dependent methyltransferases (c.66), and P‐loop containing nucleotide triphosphate hydrolases (c.37) are not substrates, despite belonging to α/β proteins. This structural bias provides a strong clue toward exploring a structural determinant of GroE substrate proteins. We realized that c.66 and c.37 exhibit the typical 3‐layer α/β/α sandwich architecture [31], and thus their shape is double wound (α helices on both sides of a β sheet). By contrast, c.3 is a 3‐layer β/β/α sandwich, and c.1 possesses only one α layer around a β sheet barrel. These two folds partially resemble the single‐wound shape (α helices on only one side of a β sheet). Even in the double‐wound cases, a small single‐wound motif would be partially found in the structure. This observation suggests that we could identify a common structural feature of the GroE substrates, ideally a structural motif, when focusing only on their local structures, instead of the entire folds.

In this study, we compared structural pairs of the GroE substrates in class IV [25, 26] [(i, ii) in Fig. 1], and constructed a library of substructures onto which hydrophobicity patterns were projected (iii). To conduct a comprehensive structure comparison, we employed MICAN, a structural alignment tool with three comparison modes [32]. Other than the standard structural alignment mode that takes the order of secondary structural elements (SSEs: α helices and β sheets) along the protein sequence into account [sequential (SQ) mode] [33], MICAN can align structures while bypassing the connectivity of SSEs [rewiring (RW) mode]. Additionally, it can ignore both the connectivity and orientation of SSEs [rewiring and reverse (RR) mode]. This characteristic feature of MICAN enables it to compare only the spatial locations of SSEs, and thus facilitates the detection of weak structural similarity beyond the borders of structural classes and folds [34]. Our library was used to optimize the discrimination of GroE substrates and negative samples (iv). As a result, we identified a couple of substructures, among which at least one hits on the structures of GroE substrates but not the negative samples (v). To date, for the GroE substrates in class III (or IV), most discriminators have been developed based on protein sequences [35, 36, 37, 38]. This is the first time that structural information has been explicitly integrated into the prediction system. Our discrimination was applied to samples that were not used in the learning process (vi). In addition, false positive predictions were experimentally validated (vii). We found new GroE obligate substrates, thereby indicating the merit of the prediction method and the utility of the idea of common substructures.

**Fig. 1:**
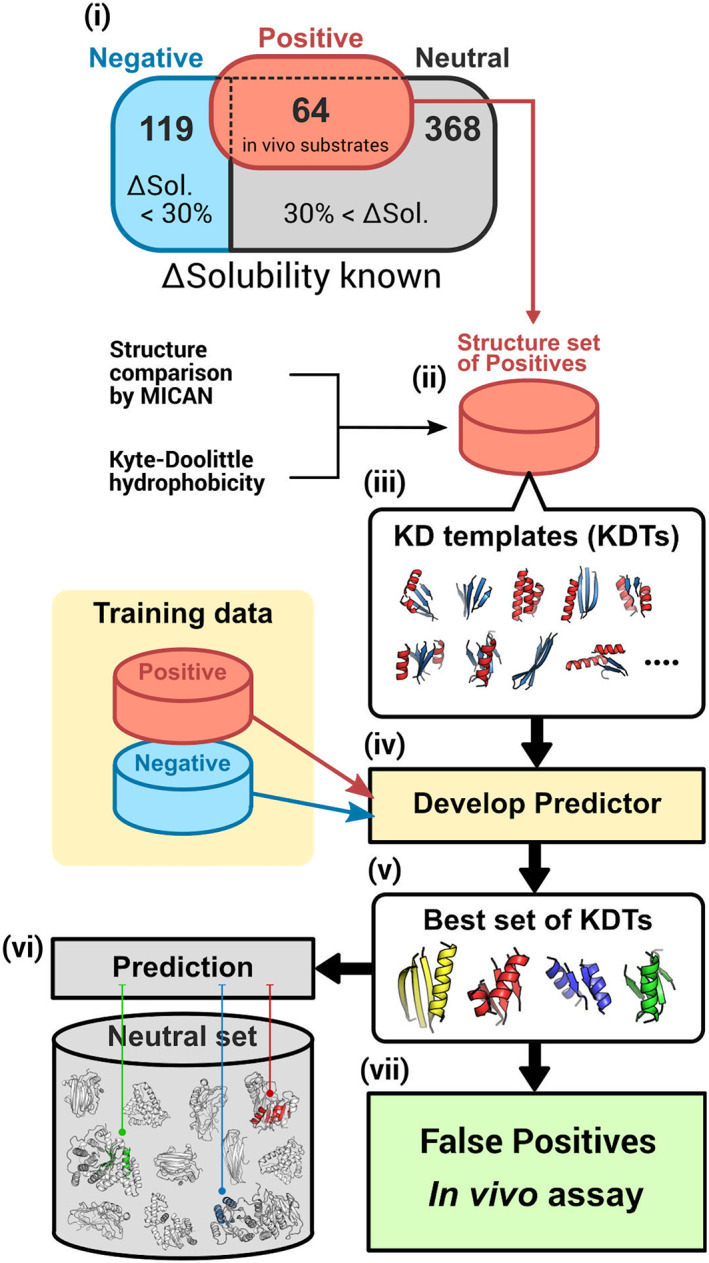
Outline of the research. (i) Dataset. (ii) Structure comparison by MICAN. (iii) Definition of KDTs. (iv) Benchmark. (v) Selection of the best KDTs. (vi) Prediction using neutral samples. (vii) Validation of false positives.

## Results and Discussion

### Development of GroE substrate predictor using a small number of substructures with hydrophobicity patterns

We prepared three datasets from the available protein structures (Table S1). GroE obligate substrates (class IV) are the positive samples (64 proteins) [25, 26]. Aggregation‐prone proteins [ΔSolubility (ΔSol: solubility gain under GroE environment) < 30%] [7] are the negative samples (119 proteins). The neutral samples (368 proteins, Materials and Methods) were used to evaluate the correlation of ΔSol and the positive prediction rate (i in Fig. 1). Assignments of datasets, based on the previous experimental results, were shown in the ‘positive/negative/neutral’ column in Table S1.

For the positive samples, MICAN conducted pairwise structure alignments with three (SQ, RW, RR) comparison modes (ii in Fig. 1) [7, 32], and extracted common substructures composed of 3 ~ 8 SSEs (excluding loops). We overlaid Kyte‐Doolittle (KD) hydrophobicity on the substructures [39], to define the KD templates (KDTs, iii). The set of KDTs formed a KDT library. When various hydrophobicity patterns were observed in similar substructures, we defined multiple KDTs using an identical (representative) substructure with different hydrophobicity patterns (Materials and Methods). Therefore, the total number of KDTs in the libraries depends on the similarity threshold of the hydrophobicity patterns. If the ideal common KDT for GroE obligate substrates existed, then that KDT would only be present in all positive samples, and absent in all negative ones. However, we could not find such a KDT. Therefore, we tried to use a union of KDTs. This system will predict the GroE obligate substrate if at least a KDT in a union is present in the structure. In the development of this predictor (iv in Fig. 1, Materials and Methods), we exhaustively optimized the comparison mode (SQ, RR, RW), the number of KDTs (up to 10), and the matching degrees of hydrophobic patterns [from rough (2) to strict (0)]. To estimate the performance, we employed the jack‐knife test and the linear programming scheme [Solving Constraint Integer Programs (SCIP)] [40] so that a union maximizes the Matthew's correlation coefficient (MCC) [41] in the discrimination (Materials and Methods).

**Fig. 2:**
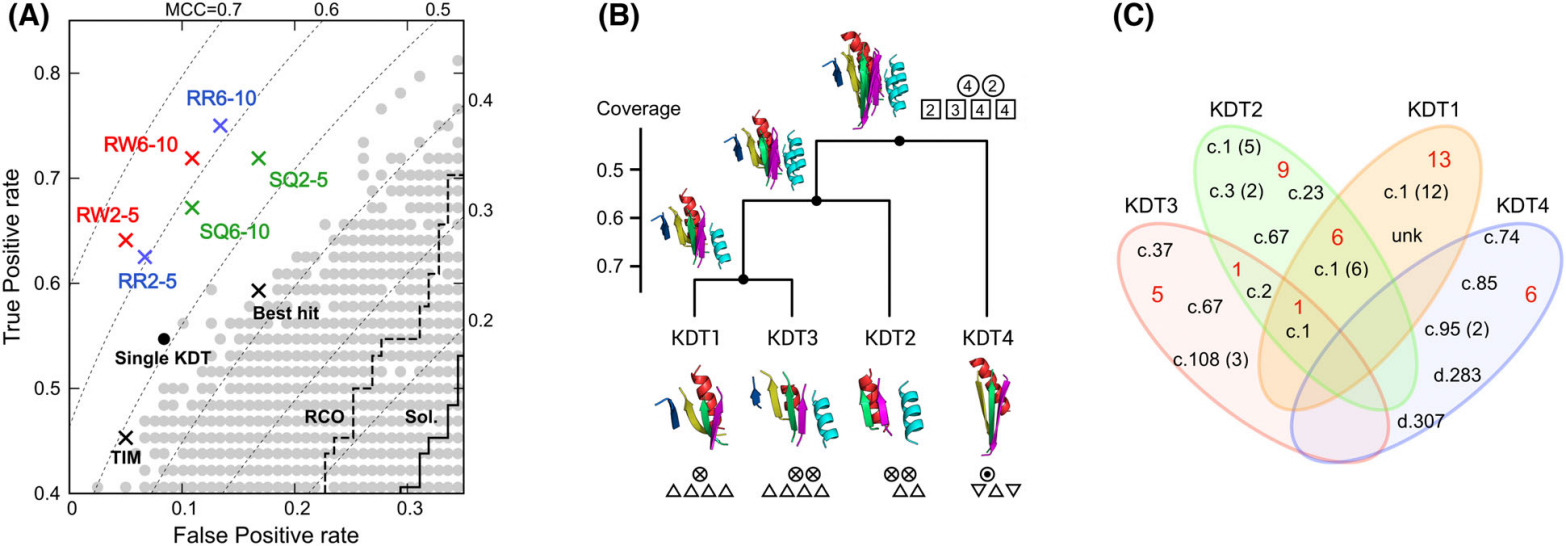
Development of GroE obligate substrate predictor. (A) Roc plot (Table S2). Points in the top left corner are better. We employed the jack‐knife procedure to evaluate the performance. Results of predictors using SQ, RW, and RR alignments are shown in green, red, and blue crosses, respectively. The figures are the number of KDTs used in the predictor (2–5 KDTs and 6–10 KDTs). For each category (cross‐section of three alignment methods and large/small number of KDTs), only the best result is shown. Gray dots are points for single KDTs, and the black one is the best. The other reference predictors are TIM (all structures adopting TIM β/α barrel fold are positives), best hit (prediction follows the attributes of the best hit), RCO (suggesting positives in the descending order of the relative contact order), and Sol (suggesting positives in the ascending order of solubility under the GroE absent conditions). MCCs are shown by the dotted lines. (B) Similarity of KDTs in the RW predictor. The KDT structures are indicated at the bottom with TOPs cartoons [44]. The left and right α helices are colored red and cyan, respectively. β strands are colored blue, yellow, green, and magenta from left to right. The orientations of SSEs in TOPs are up [dot in a circle (α); triangle (β)] or down [cross in a circle (α); inverted triangle (β)]. Structures superimposed by the MICAN RW mode are shown near the ancestral nodes of the dendrogram. All KDTs are superimposable by the MICAN RR mode (top panel). The structures are projected on the TOPs cartoon of 2α4β, and the number of corresponding SSEs is in the top right panel. (C) Role of each KDT presented in a four‐set Venn diagram. True positives detected by each KDT are shown by SCCS. Numbers in parentheses are multiple true positives in a fold. Red figures are the total number of true positives in the box.

The Receiver operating characteristic (Roc) plot of the parameter scanning results is shown in Fig. 2A (see also Tables S2 and S3), in which true positive and false positive rates are shown in the ordinate and the abscissa, respectively. Plots in the upper left part indicate better performance, in which KDTs are more exclusively included in GroE substrate structures. When we only use a single KDT, the discrimination power is poor (0.5 MCC at most) for any degree of hydrophobic pattern and any comparison mode (gray dots). We tested the unions from 2 to 10 KDTs, and the best performance was selected from the groups of 2–5 and 6–10 KDTs. The union of 4 KDTs with the RW mode (at a moderate 0.65 hydrophobicity threshold) performed the best (0.644 MCC, see ‘RW’ column in Table S1 for the prediction results) among all tested groups (Table S2). This predictor (the RW predictor, Table S4) performed significantly better than the best one using the SQ mode (< 0.6 MCC), indicating that ignoring the connectivity of SSEs is an efficient strategy to match KDTs with GroE substrate structures. Actually, among the 41 true positives detected by the discriminator, 11 GroE obligate substrates were identified by only the RW alignment mode (Table S5). As compared with the groups employing the 5–10 KDTs, the union of 4 KDTs performs well. This means that, instead of using many KDTs, a small number of substructures are sufficient to identify most GroE obligate substrates. When we evaluated 4 KDTs of the RW mode, ignoring the KD hydrophobic pattern (cut‐off threshold of 2), its performance decreased (0.604 MCC). Considering the hydrophobic pattern is also effective. Actually, the RW predictor accepted a number of structures (alignments against KDTs) at the structural comparison and examined their hydrophobic pattern in more detail (Fig. S1, Table S5). It should be empathized that both the structural and hydrophobic criteria fill each role (Text S1). We evaluated the TIM‐barrel criterion, in which the TIM β/α barrel fold was automatically predicted as positive, as well as the α/β criterion (α/β proteins are positive). Additionally, we tested the best‐hit method (following the attributes of the closest BLAST hit [42], Table S3), the RCO method (predicting positives in the descending order of relative contact order), and the Sol method (predicting positives in the ascending order of solubility, [7]). In the results of the best‐hit method, the GroE dependency of closest homolog is distinct to that of the query (e.g., JW1670 and JW2781 in Table S3), and it has been known that a small number of amino acid substitutions affect the GroE dependency [20, 43]. This indicates that relying only on the homology or sequence similarity is ineffective. The results of compared methods were clearly worse (Fig. 2A and Table S2), demonstrating that the RW predictor works well.

The benchmark system can perform a more complicated prediction, using a mixture of different comparison modes (Materials and Methods). The mixed 5 KDTs of the RW and RR modes at the 0.6 hydrophobicity cut‐off (the RW + RR predictor, Table S6) performs (0.646 MCC, see ‘RWRR’ column in Table S1 for the prediction results), as well as the RW predictor (Table S2). We investigated these two cases further but mainly described the simple RW predictor.

### The best discriminator is the 2‐layer 2α4β sandwich

We examined the KDTs (KDTs 1–4) used in the RW predictor. The MICAN comparison revealed that they are remarkably similar and superimposable on the 2‐layer 2α4β sandwich (hereafter, 2α4β). The dendrogram of the structural similarity of 4 substructures, in which MICAN used the RW mode and the similarity score (vertical axis) was defined by the coverage (the rate of overlap regions in the alignment), is shown in Fig. 2B. Structure of each KDT is illustrated in the bottom with TOPs cartoon, in which arrangement of α helices and β strands are schematically represented by circles and triangles, respectively [44] (see the legend of Fig. 2B). KDTs 1–3 are composed of a parallel β sheet and one or two helices, and they are alignable with at least 0.43 coverage. KDT4 has a different shape comprising an antiparallel β sheet with rather long strands and a helix. The worst coverage of KDT4 is 0.32 against KDT2, but if the RR mode is applied, the score is 0.55 and all KDTs can be superimposed (top right structures in Fig. 2B). The original domain structures of KDTs are summarized in Table S4. Interestingly, all KDTs were derived from the common substructure between the TIM β/α barrel and the other α/β proteins. Note that 2α4β is ubiquitous and the most abundant substructure in the structural domains [34, 45, 46, 47]. Targeting this structural pattern should be an effective strategy for GroE to maximize its assistance of numerous proteins. By contrast, this prediction method requires some portion of α helices and β strands in principle. Therefore, it is difficult to predict obligate substrates whose structure is mainly composed of α helices or β strands (Text S1, Table S5). This is the limitation of the method, and other features are required to detect such obligate substrates. The 5 templates (KDTs 5–9) used in the RW + RR predictor (Table S6) are also superimposable on 2α4β (Fig. S2).

### GroE obligate substrates identified as TIM β/α barrel, α/β proteins, and outliers

Although 4KDTs show structural similarity to some extent, each KDT has its own specific role in substrate discrimination. The classification of true positives (41 samples) detected by each KDT is depicted in a Venn diagram in Fig. 2C. KDT1 is the main, special template that only identifies TIM β/α barrels (c.1). KDT1 detects half of the true positives, and wrongly scores two negative samples (both TIM β/α barrels). KDT2 assists KDT1. It detects five TIM β/α barrels that KDT1 missed. In addition, KDT2 identifies other folds [NAD(P)‐binding Rossmann‐fold domains (c.2), FAD/NAD(P)‐binding domain (c.3), Flavodoxin‐like (c.23), PLP‐dependent transferase‐like (c.67)], among which some (c.3, c.67) may deviate from the typical double‐wound architecture. KDTs 1 and 2 detect three‐quarters of the true positives (30 samples), with 7 samples multiplied identified by both. KDT3 finds the other type of α/β proteins, including typical double wounds [P‐loop containing nucleoside triphosphate hydrolases (c.37) and HAD‐like (c.108)]. Two true positives are overlapped by KDT2. In contrast to the above three KDTs, KDT4 shows different preferences. It detects not only a couple of α/β proteins that contain an antiparallel β sheet but also α + β proteins. The true positives detected by KDT4 are unique, and cannot be identified by KDTs 1–3. As the TOPs cartoon shows (bottom of Fig. 2B), its SSE spatial arrangement is distinct from the others, thus facilitating the identification of outliers. An analysis of the role of each KDT indicated that GroE obligate substrates are divided into two categories: those adopting TIM β/α barrels or some α/β proteins involving a parallel β sheet detectable by KDTs 1–3, and those adopting other α/β proteins or α + β proteins involving an antiparallel β sheet detectable by KDT4. The alignments of true positive samples and KDTs are indicated in Data [Link], [Link]. The role allotments of the KDTs in the RW + RR predictor (KDTs 5–9) are schematically similar to those of KDTs 1–4 (Text S1, Fig. S3): They are the main detectors of the TIM β/α barrel fold (KDT5) and its supporter (KDT6), as well as the detectors for α/β proteins mainly composed of parallel β sheets (KDT7), and other α/β proteins and α + β proteins (KDTs 8 and 9).

### Positive prediction rate of GroE substrate correlates with ΔSol

**Fig. 3:**
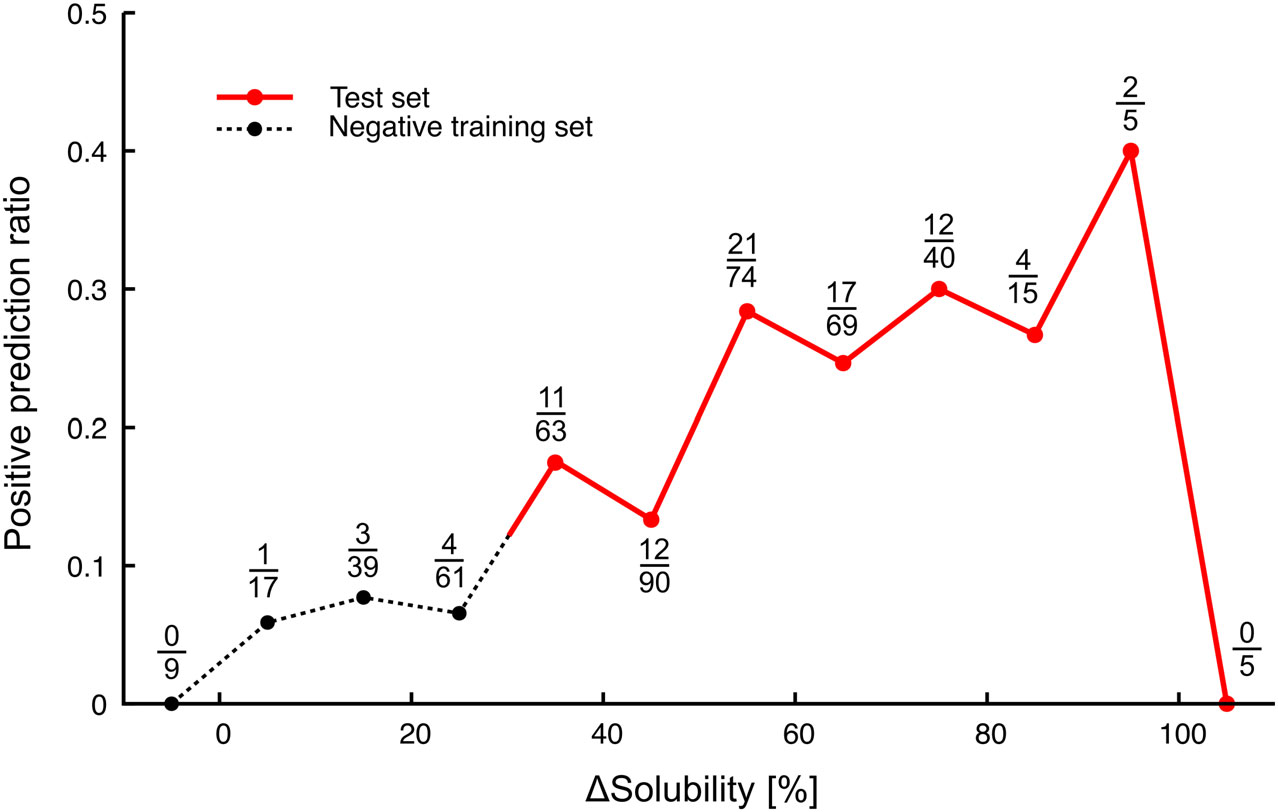
Positive prediction ratio along the ΔSol (Solubility). Positive prediction ratios were calculated for 10% bins of ΔSol. The numbers of positives and total samples in the bin are presented as fractions. Ratios for the neutral and negative samples are plotted in red and dotted lines, respectively. The 126 proteins plotted under 30% ΔSol are 119 negative samples and 7 neutral samples in class I‐III (Materials and Methods, Table S1) [21]. For some proteins, ΔSol was less than 0% or greater than 100%. This is simply due to the experimental error. Since the data were obtained from a comprehensive manual analysis, experimental errors were inevitable, even if we conducted multiple experiments and the values were averaged.

ΔSol is the solubility difference for a protein in environments with and without GroE *in vitro* [7]. When ΔSol is large, it is reasonably considered that GroE recognizes the protein and precludes its aggregation. In fact, most class IV substrates have low solubilities in the absence of any chaperones and high solubility in the presence of GroE (i.e., high ΔSol values), and we identified several GroE obligate substrates by referring to ΔSol [26]. Accordingly, ΔSol is an appropriate measure to evaluate whether the predicted proteins are truly *in vivo* GroE obligate substrates. We applied the RW predictor to 368 neutral samples (Materials and Methods) that were not employed in the learning process. The ratio of positive prediction for each bin of ΔSol (10% width), as well as the positive ratio in negative samples, is shown in Fig. 3. We noticed that the ratio correlates with ΔSol. For the samples of ΔSol > 30% (see Fig. 3 legend), the correlation coefficient is almost zero, but it is obviously attributed to the data of ΔSol > 100% (outlier). When removing statistically unreliable data with small denominators (< 10 samples in the bin), the coefficient is 0.75. For all data including ΔSol < 30%, except for the bins with fewer than 10 samples, the coefficient is 0.91. The positive prediction rate linearly increases according to the gain of ΔSol, indicating that the prediction reflects the essential nature of ΔSol, and demonstrating that the prediction is reliable (see Text S1, Fig. S4 for the RW + RR predictor).

### Experimental examination of false positives: More than half are novel GroE obligate substrates

**Fig. 4:**
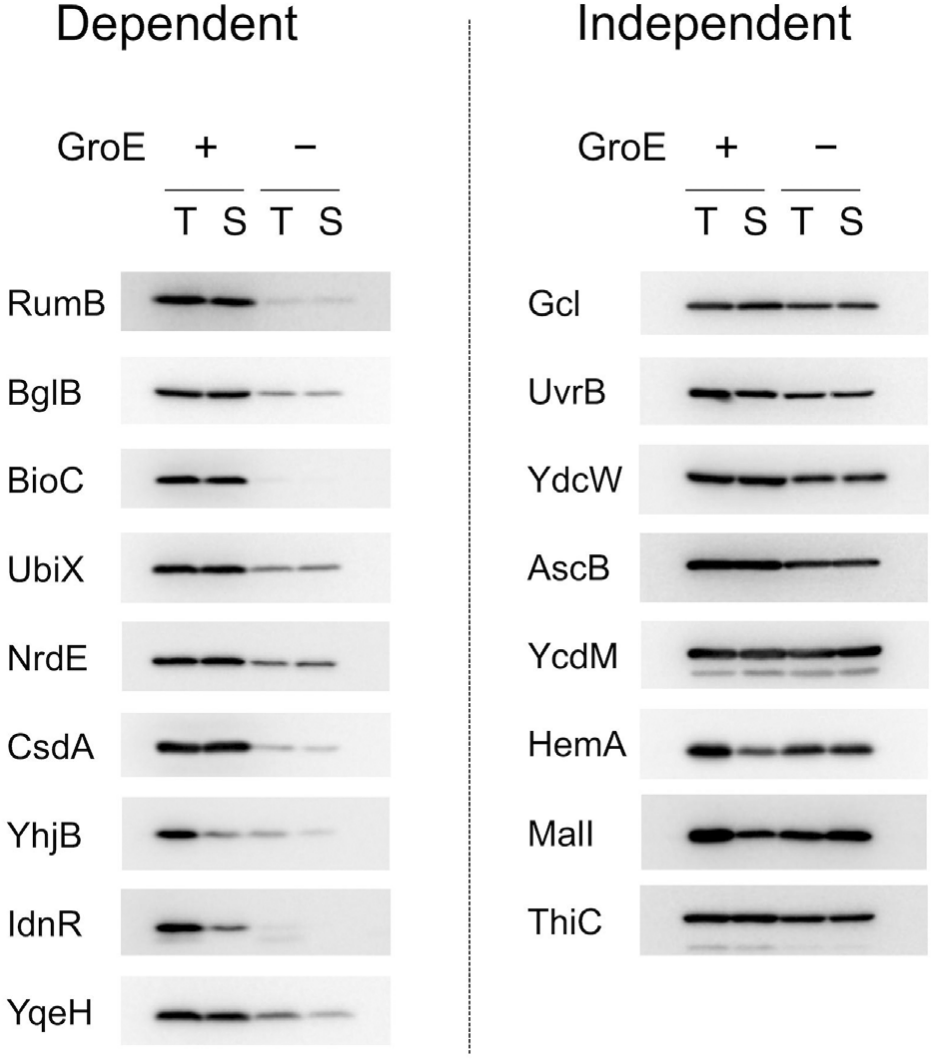
Evaluation of GroE dependencies *in vivo*. Each protein was expressed under GroE+ and GroE− conditions and analyzed by a centrifugation‐based method (see Materials and Methods). ‘T’ and ‘S’ represent the total and supernatant fractions, respectively. The proteins with decreased intensities in S of GroE− as compared to T of GroE− or decreased intensities in T of GroE− as compared to T of GroE+ were determined to be GroE obligate substrates *in vivo*.

The RW and RW + RR predictors incorrectly evaluated 17 negative samples as positives (false positives). Their domains detected by KDTs were categorized into 12 folds (Table 1). While 5 folds among them are popular or already exist in the identified positives, the rest are only seen in the negatives. Because the negative samples were only defined by ΔSol, their predictions may not be truly wrong; i.e., the false positives could still be GroE obligate substrates (class IV). Therefore, we experimentally evaluated the *in vivo* dependencies of their folding on GroE, by using GroE‐depleted cells (GroE−) [26]. If the evaluated protein is a GroE obligate substrate, its solubility should be low, or its expression should be severely diminished under GroE‐depleted conditions, where the substrates fail to fold correctly and are then targeted by proteases such as Lon protease [25, 26, 48]. Their solubilities and expression amounts in the GroE‐depleted cells (GroE−) were compared against those in the GroE‐expressing cells (GroE+). We conducted the analysis twice as replicates, and only the proteins that showed clear GroE dependences in both replicates were defined as the obligate GroE substrates. The results are summarized in Table 1 and Fig. 4. As a result, 9 proteins were newly identified as GroE obligate substrates, and 8 as GroE independent folders.

**Table 1:** List of false positives and their experimental validation. RW: KDTs (1–4) detecting the protein; RW+RR: KDTs (5–9) detecting the protein; PDBid: Template structure of homology modelling is labelled by *. Matched domain with KDT is shown in bold; P/N; number of folds in positive and negative datasets.

JW ID	Gene	Protein	MW (kDa)	GroE dependency	RW	RW + RR	PDBid (*template)	SCCS	P/N
JW0843	rumB	23S rRNA m(5)U747 methyltransferase	41.9	+	KDT3,4		2jjqA*	**c.66.1**	0/3
JW3699	bglB	Cryptic phospho‐beta‐glucosidase B	53.1	+	KDT1		2xhyA*	**c.1.8**	28/7
JW0760	bioC	Predicted methyltransferase, enzyme of biotin synthesis	28.3	+		KDT7	3bkwA*	**c.66.1**	0/3
JW2308	ubiX	3‐octaprenyl‐4‐hydroxybenzoate carboxy‐lyase	20.7	+		KDT6	1sbzA*	**c.34.1**	0/1
JW2650	nrdE	Ribonucleoside‐diphosphate reductase 2, alpha subunit	80.4	+		KDT6,7	1peqA*	a.98.1_**c.7.1**	0/3
JW2781	csdA	Cysteine sulfinate desulfinase	43.2	+		KDT9	1kmkA*	**c.67.1**	5/1
JW3488	yhjB	Predicted DNA‐binding response regulator in two‐component regulatory system	22.6	+		KDT7	4gvpA*	**c.23.1**_a.4.6	1/5
JW4221	idnR	DNA‐binding transcriptional repressor, 5‐gluconate‐binding	37.5	+		KDT5,8	3kjxA*	**c.93.1**_a.35.1	0/2
JW5454	yqeH	Conserved protein with bipartite regulator domain	24.3	+		KDT6	4gvpA*	**c.23.1**_a.4.6	1/5
JW0495	gcl	Glyoxylate carboligase	64.7	−	KDT2,3		2panA	**c.36.1**_c.31.1	0/5
JW0762	uvrB	Excinulease of nucleotide excision repair, DNA damage recognition component	76.2	−	KDT2		2d7dA*	**c.37.1**	2/9
JW1439	ydcW	2‐ketoacid reductase	50.8	−	KDT2		1wndA	**c.82.1**	0/2
JW2686	ascB	Protein required for maturation of hydrogenase 3	53.9	−	KDT1,3	KDT5	4f66A*	**c.1.8**	28/7
JW0997	ycdM	Monooxygenase ycdM	42.2	−		KDT5	1nqkA*	**c.1.16**	28/7
JW1201	hemA	Glutamyl tRNA reductase	46.3	−		KDT5,7,8	1gpjA	d.58.39_**c.2.1**	4/10
JW1612	malI	DNA‐binding transcriptional repressor	36.6	−		KDT5,8	2pugA*	a.35.1_**c.93.1**	0/2
JW3958	thiC	Thiamin (pyrimidine moiety) biosynthesis protein	70.8	−		KDT5	3epoA*	unk	−

**Fig. 5:**
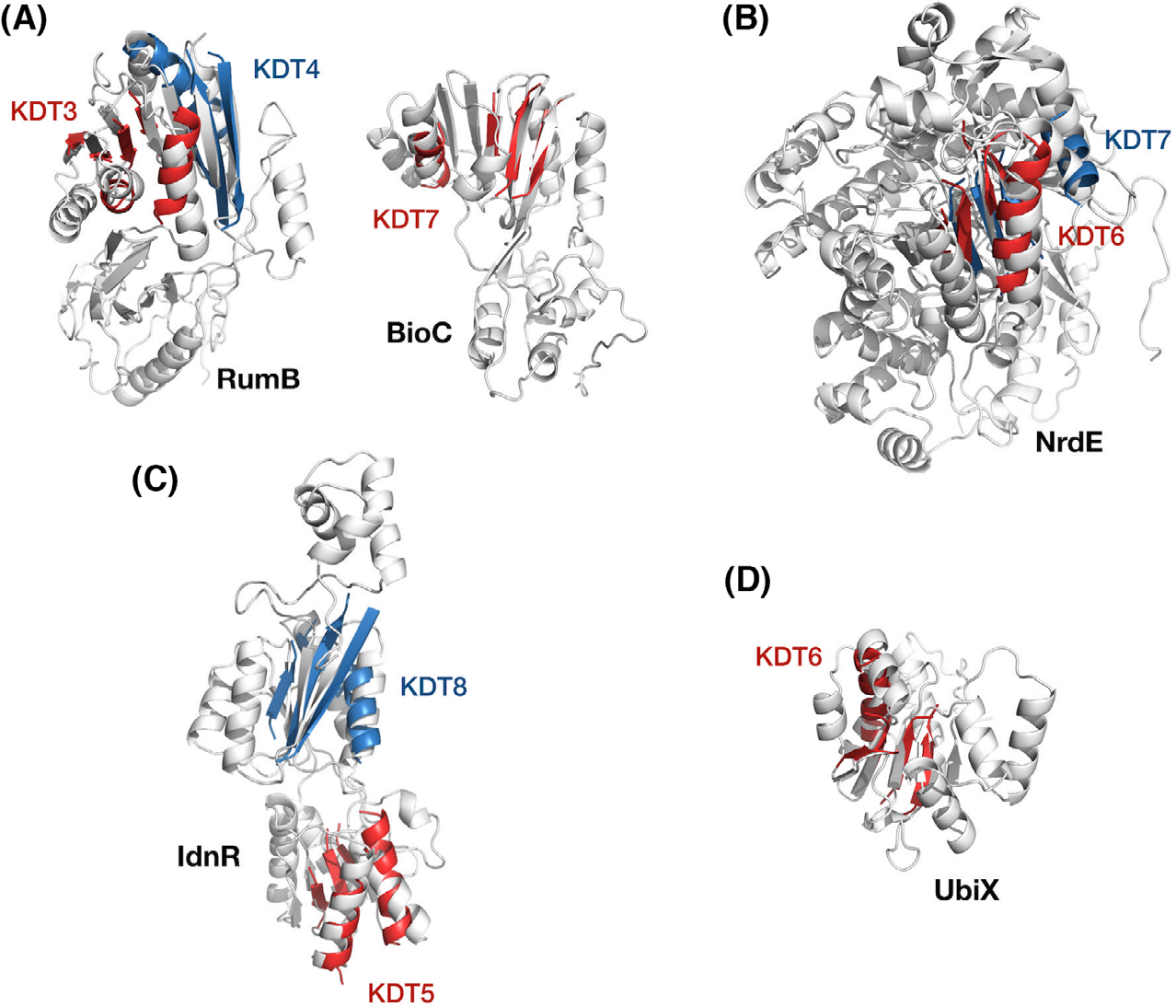
Structures of GroE obligate substrates with the folds appearing in class IV for the first time. Match regions and KDTs are superimposed. (A) RumB and BioC (c.66: S‐adenosyl‐l‐methionine‐dependent methyltransferases). (B) NrdE (c.7: PFL‐like glycyl radical enzymes). (C) IdnR (c.93: Periplasmic binding protein‐like I). (D) UbiX (c.34: Halotolerance protein Hal3).

Four folds appeared in class IV for the first time (Fig. 5). Note that our method can detect the ‘new fold’ substrates that were not included in the positive learning set because it only considers the local structure. We preferentially accepted the experimental structures, but in all four cases, they were absent. Then, the homology models were built and examined (the Protein Data Bank (PDB) id column in Table 1). RumB (modeled from PDB_ID; 2jjqA [49]) and BioC (3bkwA) both adopt the S‐adenosyl‐l‐methionine‐dependent methyltransferase fold (c.66), which shows strongly negative enrichment in GroE substrates (Fig. 5A) [25]. NrdE (c.7: PFL‐like glycyl radical enzymes, 1peqA [50]) is the largest GroE‐dependent protein (MW: 80.4 kDa) among all class IV proteins identified so far (Fig. 5B) [25, 26]. We note that the size of NrdE cannot be encapsulated in the GroEL–GroES cavity, which accommodates ~ 60 kDa proteins at the maximum [51]. The result suggests that the cavity might be not essential for some *in vivo* substrates, as already shown for some *in vitro* stringent substrates, such as aconitase (82 kDa) [52]. IdnR (c.93: Periplasmic binding protein‐like I, 3kjxA, Fig. 5c) and UbiX (c.34: Halotolerance protein Hal3, 1sbzA [53], Fig. 5d) were also identified (in SCOP2 [54], the Periplasmic binding protein‐like I fold was integrated into the Flavodoxin‐like fold). In addition to the rare folds in the positives, proteins adopting popular folds were also identified. Regarding the PLP‐dependent transferases‐like fold (c.67) appearing four times in the positive proteins, only CsdA (1kmkA [55]) was in the negative group but was confirmed to be positive. Consequently, all proteins adopting the fold in the learning dataset are classified into class IV. Although the most popular fold in the positives is the TIM β/α barrel fold (c.1, 28 positives), 7 barrel‐containing proteins remained in the negatives, and three of them were detected by the predictors. While BglB (2xhyA [56]) was evaluated to be a GroE‐dependent substrate, AscB and YcdM were not. Other GroE obligate substrates are AegA (c.3: FAD/NAD(P)‐binding domain, 2vdcG [57]), and YhjB and YqeH (c.23: Flavodoxin‐like, both 4gvpA [58]).

For the newly identified samples, we examined the regions of KDT hits on the structures and found that multiple KDTs hit on similar regions in some cases (Table 1, Text S1, Fig. S5). However, the relationship between multiple KDT hits at the localized region and the prediction confidence is currently uncertain. We observed many single KDT hits in the true positive predictions (Table S1) and multiple KDT hits in the GroE independent folders (AscB, Gcl, and HemA: especially, three KDTs hit a very similar region of HemA). Therefore, estimating the prediction reliability is one of the significant future issues.

It is reasonable to anticipate that the refinement of the learning dataset will improve the method. We added 9 new GroE obligate substrates into the positive samples and conducted the structural comparison. Originally, MICAN with the RW mode produced 284 structural clusters. A KDT library adopting 0.65 hydrophobicity threshold comprises 322 KDTs. When taking new GroE obligate substrates into account, we obtained 374 structural clusters and 413 KDTs. In both cases, libraries involved almost 90 new templates. We evaluated the performance of each new KDT and found that a couple of KDTs can detect positives that could not be identified by the RW and the RW + RR predictors (Table S8). Although it is uncertain whether these KDTs are selected through the jack‐knife test, gaining a variety of KDTs is promising. Another issue is the reliability of negative samples, in which some proteins were revealed to be misclassified by the experimental evaluation of GroE dependency *in vivo*. Because the examination of all negative samples by the experiment is unrealistic, we applied it to remained 4 proteins adopting the TIM β/α barrel fold in the negative samples. Surprisingly, it was found that 3 samples were the GroE obligate substrates (Fig. S6). We consider that relying on low ΔSol was the best way to define the negative samples if using data from the reconstituted cell‐free system, but further correction is required.

### Substructures mapped on the folding core of TIM β/α barrels

**Fig. 6:**
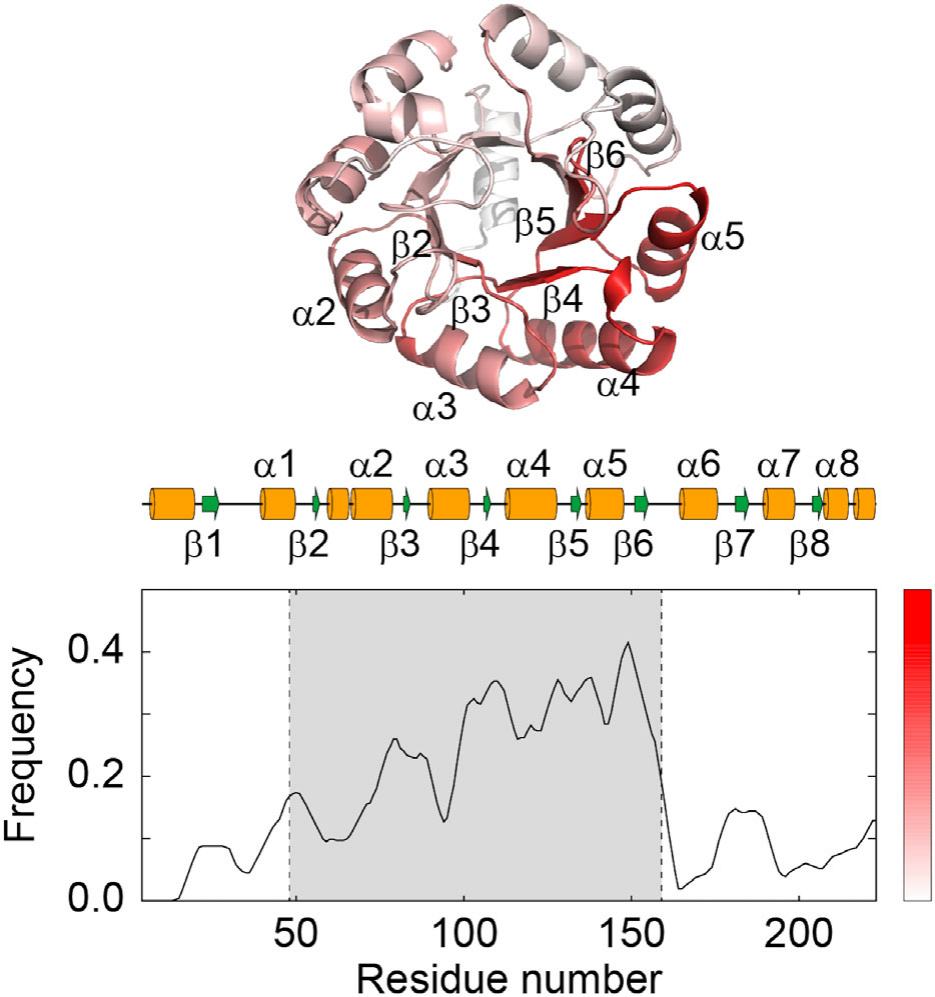
Match regions of substrates adopting the TIM β/α barrel fold with KDTs 1 and 2. KDT1 and KDT2 identified 19 and 12 TIM β/α barrels, respectively. Each structure was aligned to the reference structure (indole‐3‐glycerol phosphate synthase (PDB_ID: 2c3zA) [70]. We counted how many times each residue matched with KDTs. The average match ratio over the neighboring 15 residues was calculated and shown in the lower panel. The folding core is highlighted in gray. The α helices and β strands in the reference structure are drawn as orange cylinders and green arrows, respectively. In the upper panel, the reference structure is illustrated, in which the higher match frequency is colored red (the right bar).

Our method only evaluates substructures in the folded proteins. By contrast, GroE recognizes unfolded or partially folded proteins, instead of structured ones. The substructures are apparently unconnected with the substrate recognition mechanism of GroE. Since almost half of the positive samples adopt the TIM β/α barrel fold (28 among 64 samples [21, 25, 26]), investigating the matches of KDTs with the fold may provide some clues for the substrate recognition mechanism of GroE. We examined the structural parts of the TIM β/α barrel folds aligned with KDTs. Fig. 6 indicates the statistics of the matched parts. KDTs 1 and 2 matched with the regions around the middle part of the TIM β/α barrel fold, instead of the N‐ and C‐terminal parts. A similar plot was obtained for KDTs 5 and 6 in the RW + RR model that correspond to KDTs 1 and 2, respectively (Fig. S7). Previous hydrogen‐exchange experiments investigating the folding core of the TIM β/α barrel fold as an intermediate state revealed its location in the middle part of the amino acid sequences (2β–6β) [59, 60]. This location schematically corresponds to the frequently matched parts of the TIM β/α barrel fold with KDTs, and suggests a speculative scenario for the substrate recognition mechanism of GroE, as follows. A substrate protein starts to fold after its synthesis, and initially forms a folding core schematically similar to 2α4β. GroEL tends to recognize the folding core to prevent the protein from aggregating. This hypothesis can reasonably explain the structural bias seen in the GroE substrates, since many partly contain 2α4β (Fig. 2B), the most abundant substructure found in natural proteins. However, the structural match results only imply that KDTs may associate with the folding core. To clarify the binding mechanism of GroEL and its substrates, more investigations of the folding process of GroE substrate proteins and their interactions with GroEL are needed.

## Conclusions

We developed a prediction method for GroE obligate substrates, using a small number of substructures with hydrophobicity patterns as probes. The method is more accurate, as compared to the homology‐based or solubility‐data methods. The substructures are superimposable on the 2α4β, the most abundant small structural pattern in known protein folds. For chaperones, the strategy targeting this motif is very effective, because GroE can assist many proteins that share it. We experimentally evaluated 17 false positives in the prediction and found 9 new GroE obligate substrates (they are correct predictions). The success of the prediction provides novel insight into substrate recognition by GroEL. The substructures were found around the folding core of TIM β/α barrel folds, suggesting that GroEL recognizes the substructure in an intermediate state during the folding. Several proteins, including other chaperones [61], ubiquitin ligases [62], protein kinases [63, 64], etc., recognize multiple and diverse proteins as substrates. Accordingly, we anticipate that their substrates possess ambiguous sequences or structural motifs. The present idea of common substructures is promising to distinguish such substrates from other proteins, and is worthwhile for further examination in the near future, because more reliable substrate structure models are available [65].

The prediction method is downloadable from GitHub (https://github.com/ShintaroMinami/gELpred).

## Materials and methods

### Development of predictor

#### Dataset

We prepared three groups of *E. coli* proteins, corresponding to positive, negative, and neutral samples. The positive samples are experimentally determined GroE obligate substrates (Class IV) [25, 26]. For negative and neutral samples, solubilities under GroE absent and present conditions were measured [7], and the difference (gain of solubility by GroE, ΔSol) was calculated. The ΔSol of a negative sample is less than 30%, indicating that it is still aggregation‐prone in the presence of GroE. All other samples are in the neutral group. In addition, we classified proteins in classes I, II, and III into the neutral group [21] if they are not in class IV. Some of their ΔSols are less than 30%. Note that ΔSol values have only been obtained from proteins with low solubility without chaperones (below 30%). Therefore, for proteins whose solubility was originally above 30%, there are no data available even if solubility was greatly improved when GroE was present. When the structures of the protein samples were determined with more than 80% coverage, we accepted the native structure. Otherwise, we tried to construct homology models for the samples, using PSI‐BLAST [42] and Modeler [66] with the secure 10^−10^ E‐value threshold. Models with more than 80% coverage were accepted. As a result, among 795 proteins requiring models, 64 (including 29 native ones), 119 (16), and 368 (126) protein structures were obtained for the positive, negative, and neutral samples, respectively (Table S1). The latest SCCS in SCOPe [30] was assigned to the PDB sequences [67] in the PSI‐BLAST alignment. The SCCS value of each protein was decided with a 0.01 E‐value threshold, and visual inspections if needed. For the multiple domains in Table S1, the order of the domains along the sequence and multiple appearances of the same SCCS are ignored (SCCSs are sorted in the ascending order).

#### Selection of substructures

We focused on the 64 structures of the positive samples, as we hypothesized that most GroE obligate substrates partly include a common or similar substructural pattern. To explore such substructures, we conducted an exhaustive structure comparison by MICAN that aligns two protein structures by applying one SQ [33] and two nonsequential modes [32], described as follows.

##### Sequential (SQ)

The conventional structure comparison, in which structurally equivalent regions must be aligned in the same order along the protein sequences.

##### Rewiring (RW)

A nonsequential structure comparison, in which structurally equivalent SSEs are aligned while ignoring their orders along the protein sequences. In other words, SSEs can be r‐ewired  differently from the native connectivity.

##### Rewiring and reverse (RR)

In addition to RW, structurally equivalent SSEs can be aligned in the N‐ to C‐terminal direction upside‐down. In this mode, only the space filling of SSEs is compared.

MICAN with each mode provides the best local alignment of two structures, and thus we can obtain two aligned substructures. Among the products, we first selected the candidates of common substructures (loops excluded) satisfying the three criteria below.

1. Well‐aligned. Cα RMSD of aligned region is 3 Å at most.
2. Suitable size for a super‐secondary structure. The number of SSEs is from 3 to 8.
3. Well packed. Every SSE has more than three residue contacts [Cβ‐Cβ (Cα for Gly) distance <7 Å] with at least one other SSE.

With the SQ, RW, and RR modes, 694, 928, and 1252 substructures were obtained, respectively.

Next, the selected substructures were clustered by MICAN at each mode, to remove the redundancy. For two substructures aligned by MICAN, we calculated the alignment coverage (number of aligned residues divided by the larger of the two substructures). We applied the average‐linkage clustering, employing ‘1—the alignment coverage’ as the distance. The clustering terminated at a distance of 0.2 (0.8 coverage), and 203, 284, and 370 structural clusters were, respectively, obtained by the SQ, RW, and RR modes of MICAN. The closest substructure to the cluster centroid was regarded as the representative structure that defines the backbone of the KD template (below).

#### Kyte‐Doolittle template

To characterize the structural clusters in more detail, we considered the hydrophobicity patterns. The Kyte and Doolittle (KD) index [39] was employed for this purpose. Each substructure in a structural cluster was aligned against the representative one by MICAN, and the multiple structure alignment was constructed. Based on the alignment, the hydrophobic patterns of any pair of substructures were compared. The KD indices along the sequences were considered to be vectors, and their similarity was evaluated by the normalized inner product (NIP or cosθ). The hydrophobic patterns were grouped using the average‐linkage clustering, with 1‐NIP as the distance. The clustering terminated at a given cut‐off threshold. Note that the cut‐off is an optimized parameter for the predictor development process (described later). When the cut‐off was rough [high value (maximum: 2)], the hydrophobic patterns of a structural cluster were combined, and thus were nearly ignored. On the other hand, if the cut‐off was strict [low value (minimum: 0)], the hydrophobic patterns of a structural cluster were divided into various groups, and a specific hydrophobic pattern was assigned to each type. For each hydrophobic pattern group, we chose the most frequent amino acid in each column of the alignment and mapped its KD index on the site in the representative structure. Hereafter, representative substructures with KD indices are referred to as KDTs.

#### Structure‐KDT matching profile table

**Fig. 7:**
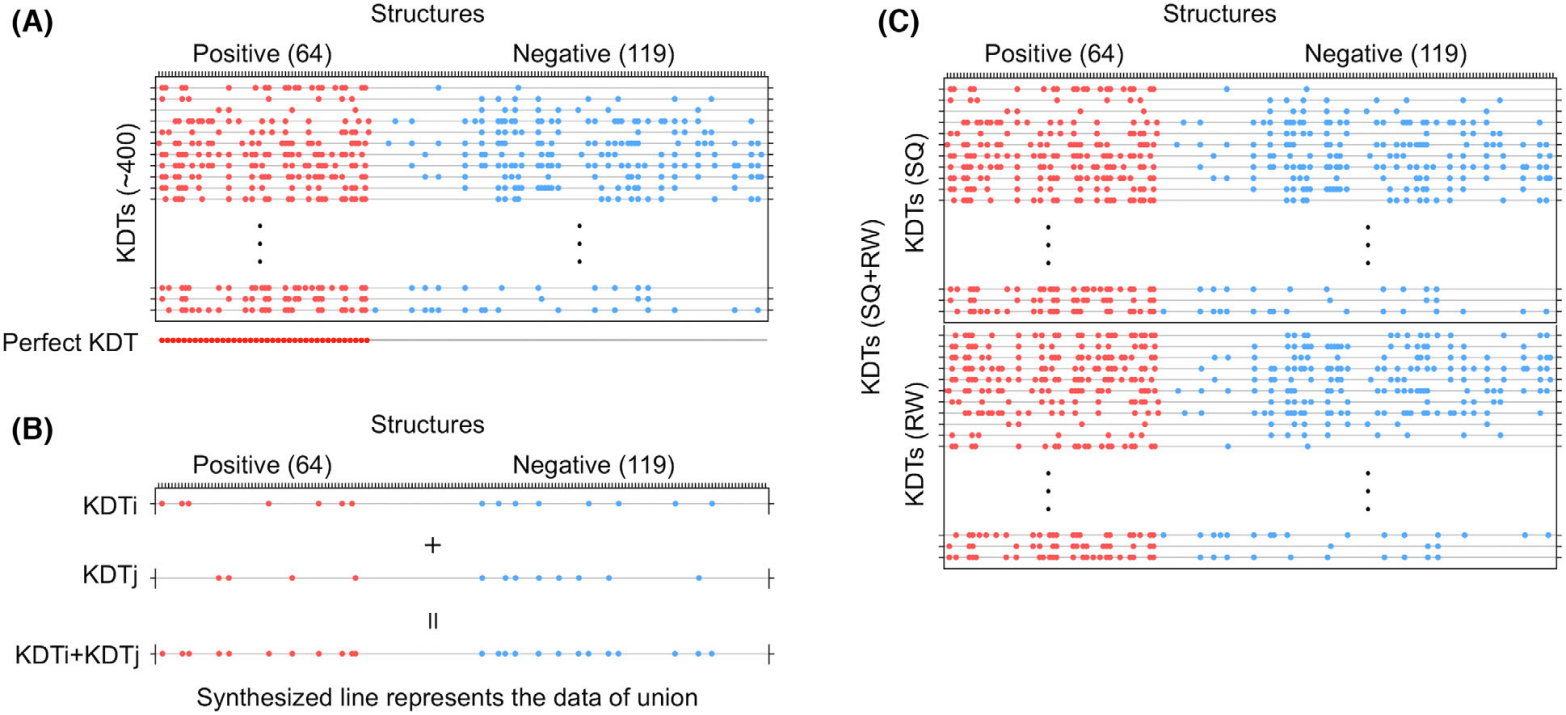
Structure‐KDT matching profile table. (A) As an example, the structures of positive and negative samples are arranged on the top, and the KDTs derived at an alignment mode and the certain hydrophobic pattern matching cut‐off are arranged on the left side. If a KDT exists in a structure, a dot is plotted at the cross‐section. Red (blue) dots indicate the match of a KDT and a positive (negative) sample. The perfect KDT, which detects all positive samples exclusively, is shown at the bottom. (B) A union of KDTs composed of KDTs i and j. A line representing the union's performance is synthesized from two lines for both KDTs. (C) A mixture of multiple tables constructed by different alignment modes. We can easily mix the structure‐KDT matching tables by simply docking the tables. In the jack‐knife test, we remove one column and process the rest with SCIP. SCIP selects the best combination of a given number of KDTs that maximizes MCC. The procedure is the same when we test a table constructed in an alignment mode.

MICAN estimated whether a KDT was found in a structure of a positive or negative sample, by applying structural and hydrophobicity criteria. First, the structures of a KDT and a positive or negative sample were compared by MICAN, using the same comparison mode as in the substructure selection. For each KDT and sample pair, the 5 best alignments were considered. We regard two structures as matched if more than or equal to 50% residues of all SSEs in the KDT were aligned, and the Cα RMSD was less than 3 Å (structure criterion). Next, for the alignments that satisfied the structure criterion, the hydrophobic pattern was evaluated. The distance (1‐NIP) of hydrophobic patterns between the aligned regions of the KDT and the sample should be within the cut‐off (hydrophobic criterion). We arranged all structures in the positive and negative samples from the left to right horizontally, and all KDTs were along the left side vertically. When a KDT was included in a sample structure, we placed a dot at the intersecting point (Fig. 7A). The illustration comprises the structure‐KDT matching profile table, in which the inclusion or exclusion of a KDT in a given sample structure is shown at a glance [68, 69]. If a perfect common KDT exists, then the KDT is only present in all positive samples but not all negative ones (the bottom of Fig. 7A). However, even the best performance of a single KDT is at an inadequate level, as its MCC is less than 0.52 (see definition later). Therefore, we considered the union (group) of KDTs, in addition to the single ones. In the system, if at least one KDT in the union exists in a sample structure, then the structure is predicted as a GroE obligate substrate.

#### Parameter optimization process

In the development of a predictor using a union of KDTs, we exhaustively scanned the structure comparison mode and parameter space and tried to find their combination that defines the best predictor. When we constructed a structure‐KDT matching profile table, we specified the structure comparison mode (SQ, RR, and RW), and the hydrophobic pattern matching cut‐off [threshold of ‘1‐NIP’ from rough (2) to strict (0)]. The constructed profile was examined for its ability to discriminate the positive and negative samples. We employed the jack‐knife test to estimate the performance, in which one column of data corresponding to one structure (positive or negative, Fig. 7A) was removed from the profile (used in the test), and the remaining data were used in the selection of KDTs. In advance of the KDT selection process, we specified the number of KDTs comprising the union (from 2 to 10). We processed the profile and the specified number of KDTs by a constraint linear programming method, Solving Constraint Integer Programs (SCIP) [40], which selected the specified number of KDTs so that its union maximized the MCC in the discrimination, defined as,

$$\mathrm{MCC}=\frac{\mathrm{TP}\times \mathrm{TN}-\mathrm{FP}\times \mathrm{FN}}{\sqrt{\left(\mathrm{TP}+\mathrm{FP}\right)\left(\mathrm{TP}+\mathrm{FN}\right)\left(\mathrm{TN}+\mathrm{FP}\right)\left(\mathrm{TN}+\mathrm{FN}\right)}},$$

where TP, FP, TN, and FN indicate the number of true positives, false positives, true negatives, and false negatives, respectively [41]. The resultant best union was applied to the removed column (structure). When at least one KDT in the union hits the structure, it is predicted as positive (GroE obligate substrate), and the accuracy of the prediction was judged as correct or incorrect. This process (remove column, select KDTs, test the union) was repeated for all structure removals. From the numbers of correct and wrong predictions, MCC was re‐calculated for the combination of comparison mode and parameters (hydrophobic pattern matching cut‐off, and number of KDTs).

Note that the data of a given union of KDTs representing their presence and absence in the structures are easily obtained, once the profile table is constructed. An example is shown in Fig. 7B, where KDTi and KDTj are selected to compose the union, and united to generate the synthesized line for the union.

#### Mixed profile

In addition to the profiles constructed by each of the SQ, RW, and RR modes at any hydrophobic pattern matching cut‐off, the optimization method can be applied to the mixture of profiles constructed by different modes. For example, when we use the profiles of the SQ and RW modes simultaneously, we simply combine them (Fig. 7c) and process them with SCIP. In the combined profile, the lines (KDTs) are composed of data of KDTs derived from the SQ and RW alignment modes. The jack‐knife procedure is applicable to the mixed profile, and the best union of KDTs can be defined in the same manner. We evaluated all combinations of different alignment modes (denoted by SQ + RW, SQ + RR, RW + RR, and SQ + RW + RR modes). Theoretically speaking, the profiles constructed at different hydrophobic pattern matching cut‐offs can be combined. However, to avoid the combinational expansion, we only mixed the profiles with the same cut‐off value.

### Plasmid construction

The genes encoding 21 proteins (17 false positives and 4 negative samples adopting the TIM β/α barrel fold) were cloned downstream of the *tac* promoter in the expression vector used in the previous work [26]. Each gene was fused with the GS linker and HA‐tag sequence, contained in the original expression vector, at its C‐terminal region for detection. All vectors were constructed by Gibson assembly with the primers listed in Table S7.

### Evaluation of GroE dependencies *in vivo*

The experimental evaluation of GroE dependency *in vivo* was performed according to the previous work [26]. MGM100, a strain in which GroE expression is controlled by arabinose, was transformed with the expression vector of a candidate protein and grown in an LB medium containing 0.2% arabinose at 37 °C to log phase. The cells were then washed with fresh LB medium and subcultured in LB medium containing 1 mm diaminopimelate and 0.2% glucose for depletion (GroE– conditions) or 0.2% arabinose as a control (GroE+ conditions). Each candidate protein was expressed under leaky conditions (without IPTG induction) during cultivation. After 3 h of cultivation, the cells were harvested, suspended in lysis buffer (20 mm Tris–HCl, pH 8.0, 100 mm NaCl, and 1 mm EDTA), and disrupted by sonication. After the disruption, the uncentrifuged total fraction and the supernatant fraction obtained by 20 000 ***g*** centrifugation were separated by SDS/PAGE. The proteins in each fraction were detected by immunoblotting with an anti‐HA monoclonal antibody (SIGMA, H9658) and an anti‐mouse antibody conjugated with horseradish peroxidase (SIGMA, A4416). The chemiluminescence signal was detected by a LAS4000 image analyzer (Fujifilm).

## Conflict of interest

The authors declare no conflict of interest.

## Author contributions

SM, TN, HT, and MO designed the project. SM, RK, and MO performed computations. TN and EU performed experiments. All authors analyzed data. SM, HT, and MO wrote the paper with input from all authors.

## Supporting information


**Data S1.** Alignments of positive samples and KDT1.

**Data S2.** Alignments of positive samples and KDT2.

**Data S3.** Alignments of positive samples and KDT3.

**Data S4.** Alignments of positive samples and KDT4.

**Table S1.** List of proteins and prediction results. Explanations of columns and symbols: Synonyms, –: no synonyms in UniProt; Sol and Sol (GroE), solubilities under GroE absent and present conditions, respectively; ΔSol, Sol (GroE)‐Sol; Class (Kerner), classes defined by Kerner *et al*. –: no class was defined; Class (Fujiwara, Niwa), classes defined by Fujiwara *et al*. and Niwa *et al*., 4: class 4 defined by Fujiwara *et al*., +: class 4 defined by Niwa *et al*., –: no class was defined; positive/negative/neutral, P: positive, N: negative, –: neutral; RW, prediction of the RW model; x: positive (at least one of KDTs 1–4 hit, see results and discussions), –: negative; KDT1 ~ KDT4; hit of each KDT; x: positive, –: negative; RWRR, prediction of the RWRR model; x: positive (at least one of KDTs 5–9 hit), –: negative; KDT5 ~ KDT9; hit of each KDT; x: positive, –: negative; Structure, structure used for the prediction, Nat: native structure, Hom: homology model; SCCS, SCOP concise classification strings.
**Table S2.** Performance of predictors.
**Table S3.** Results of the best‐hit method.
**Table S4.** KDTs used in the RW predictor. * Template was defined by the common substructure of JW1 and JW2. Their SCCSs are denoted in the SCCS 1 and 2 columns. ** Red numbers: positions of α helices.
**Table S5.** Alignment of KDTs in the RW predictor and structure of positive samples.
**Table S6.** KDTs used in the RW + RR predictor. * Template was defined by the common substructure of JW1 and JW2. Their SCCSs are denoted in the SCCS 1 and 2 columns. ** Red numbers: positions of α helices.
**Table S7.** List of primers.
**Table S8.** Performance of new single KDT. This table presents only the KDTs identifying positives that the RW and the RW + RR predictors could not detect. We selected the KDTs with one or no false positive. newTP (RW): number of true positive that the RW predictor could not detect. newTP (RW, RWRR): number of true positives that the RW and the RW + RR predictors could not detect. JW: ID of newTP (RW, RWRR). MCC: MCC of single KDT. MCC (RW): MCC of the RW predictor plus the single KDT.

**Appendix S1.** Additional information for results.
**Fig. S1.** Results of hydrophobic criterion. The match of hydrophobic pattern (1‐NIP) and the averaged KD index for the aligned regions are plotted. (a) Positive samples. (b) Negative samples. Circles and crosses are the plots of alignments that passed the hydrophobic criterion and that did not, respectively. Plot of alignments against KDT1, 2, 3, and 4 are colored orange, green, red, and blue, respectively. Diamonds are the plots for KDTs, colored in the same manner to the alignments.
**Fig. S2.** KDTs used in the RW + RR predictor.
**Fig. S3.** Role of KDTs used in the RW + RR predictor. (a) Five‐circle Venn diagram showing how many positive samples (labeled by SCCS) are detected by each KDT used in the RW + RR predictor. SCCSs are shown in black characters. Multiple hits are denoted in parentheses. Red numbers are the number of positives in each box. (b) Similarity of the roles of KDTs. The similarity of two KDTs was estimated by the preferences in the positive‐sample detection using the Jaccard index. The roles of KDTs are divided into the main TIM (c.1) predictor, the supportive TIM predictor, the α/β protein predictor, and the outlier predictor.
**Fig. S4.** Positive prediction rate by the RW + RR predictor against ΔSol
**Fig. S5.** Hit regions between RumB, BioC, and KDTs. RumB and BioC adopt the same fold (c. 66), and they are alignable. MICAN aligned both proteins. KDTs 3 and 4 hit RumB, and KDT7 hit BioC. Note that KDTs are only composed of SSEs. α helices and β strands are colored orange and green, respectively.
**Fig. S6.** Evaluation of GroE dependencies *in vivo* for four proteins adopting TIM β/α barrel fold in the negative samples (JW1492; YdeM, JW2841; HyuA, JW2884; YliK, JW5511). Each protein was expressed under GroE+ and GroE‐ conditions and analyzed by a centrifugation‐based method (see Materials and Methods). ‘T’ and ‘S’ represent the total and supernatant fractions, respectively. The proteins with decreased intensities in S of GroE‐ as compared to T of GroE‐ or decreased intensities in T of GroE‐ as compared to T of GroE+ were determined to be GroE obligate substrates *in vivo*.
**Fig. S7.** Match regions of substrates adopting the TIM β/α barrel fold with KDTs 5 and 6. KDT5 and KDT6 identified 18 and 7 TIM β/α barrels, respectively. Each structure aligned to the reference structure match of each residue against KDTs was counted (see the legend of Fig. 6). The folding core of TIM β/α barrels is highlighted in gray. In the upper panel, the reference structure is illustrated, in which the higher match frequency is colored red (the right bar).

## Data Availability

The data that support the findings of this study are available in the supplementary material of this article. Structural models used in the study are provided by the corresponding author upon request.
