# Supplementary material for: Prediction of chaperonin GroE substrates using small structural patterns of proteins
Source: FEBS Open Bio. 2023 Mar 14;13(4):779–94. doi: 10.1002/2211-5463.13590 (PMC10068320; doi:10.1002/2211-5463.13590)
Supplement: Supplementary file 6 — Appendix S1. Additional information for results. Fig. S1. Results of hydrophobic criterion. The match of hydrophobic pattern (1‐NIP) and the averaged KD index for the aligned regions are plotted. (a) Positive samples. (b) Negative samples. Circles and crosses are the plots of alignments that passed the hydrophobic criterion and that did not, respectively. Plot of alignments against KDT1, 2, 3, and 4 are colored orange, green, red, and blue, respectively. Diamonds are the plots for KDTs, colored in the same manner to the alignments. Fig. S2. KDTs used in the RW + RR predictor. Fig. S3. Role of KDTs used in the RW + RR predictor. (a) Five‐circle Venn diagram showing how many positive samples (labeled by SCCS) are detected by each KDT used in the RW + RR predictor. SCCSs are shown in black characters. Multiple hits are denoted in parentheses. Red numbers are the number of positives in each box. (b) Similarity of the roles of KDTs. The similarity of two KDTs was estimated by the preferences in the positive‐sample detection using the Jaccard index. The roles of KDTs are divided into the main TIM (c.1) predictor, the supportive TIM predictor, the α/β protein predictor, and the outlier predictor. Fig. S4. Positive prediction rate by the RW + RR predictor against ΔSol Fig. S5. Hit regions between RumB, BioC, and KDTs. RumB and BioC adopt the same fold (c. 66), and they are alignable. MICAN aligned both proteins. KDTs 3 and 4 hit RumB, and KDT7 hit BioC. Note that KDTs are only composed of SSEs. α helices and β strands are colored orange and green, respectively. Fig. S6. Evaluation of GroE dependencies in vivo for four proteins adopting TIM β/α barrel fold in the negative samples (JW1492; YdeM, JW2841; HyuA, JW2884; YliK, JW5511). Each protein was expressed under GroE+ and GroE‐ conditions and analyzed by a centrifugation‐based method (see Materials and Methods). ‘T’ and ‘S’ represent the total and supernatant fractions, respectively. The proteins with decreased intensities [file FEB4-13-779-s001.pdf]

Supporting Text and Figures

For

A method that predicts chaperonin GroE substrates using small structural patterns of proteins

Shintaro Minami [1], Tatsuya Niwa [2], Eri Uemura [2], Ryotaro Koike [1], Hideki Taguchi [2], Motonori Ota [1]

[1] Graduate School of Informatics, Nagoya University
Nagoya, Aichi 464-8601, Japan

[2] Cell Biology Center, Institute of Innovative Research, Tokyo Institute of Technology
Yokohama, Kanagawa 226-8503, Japan

Text S1

Additional information about the RW predictor

Each KDT used in the RW predictor is summarized in Table S4. Note that all KDTs were selected by the structure comparisons of the TIM β/α barrel fold (c.1) and others. Alignments of detected positive samples and KDTs are shown in Data S1-S4.

As the RW alignment mode is an enhanced version of the SQ alignment mode, the sequential alignments can also be produced by the RW mode. We examined whether the alignment was a sequential or rewiring (non-sequential) product. In Table S5, the type of alignment is summarized for positive samples. As mentioned in the "Materials and Methods" section, we considered the best 5 alignments for each KDT and structure pair. The total number of hits was 58 for 41 positive samples, and almost one-third of their matches were evaluated based on the rewiring alignment (S: 40 hits, R: 18 hits). Among the 41 true positives, 11 were only detected based on the rewiring alignment, indicating that a strategy that only considers the spatial arrangement of SSEs and ignores the connection is effective. Another strategy of the RW predictor is indicated in Table S5. The number of alignments that passed the structure criterion is 297 (23%) among 1280 alignments for 64 positive samples (4 KDTs × 5 alignments × 64 samples) . However, only 20 % of them (58/297) passed the hydrophobicity criterion. That is, the structure criterion gathered the matched structures roughly and widely, and the hydrophobicity criterion examined them in more detail. We confirmed that the hydrophobicity criterion evaluated the match of hydrophobic pattern, instead of hydrophobicity itself. For the aligned part of positive samples satisfying the structure criterion, we illustrated a scatter plot (Fig. S1a) of the averaged hydrophobicity and the distance of hydrophobic pattern from a KDT (1-NIP, see Methods in detail). The averaged hydrophobic values of KDTs (diamonds) are localized around 0.3 On the other hand, in both the aligned parts that passed the hydrophobicity criterion (circles) and that did not (crosses), plots are widely distributed from hydrophilic (-0.5) to hydrophobic (1.5) values. Two distributions of averaged hydrophobicity are not different (p-value of Wilcoxon test: 0.407). This indicates the importance of taking hydrophobic pattern matching into account in the substrate discrimination.

For negative samples, satisfying the structural criterion is rather difficult, because many structures do not contain the shape of KDTs. Among total 2380 alignments for 119 negative samples, only 199 (8%) passed the criterion. Satisfying the hydrophobicity criterion is more difficult. Only 9 alignments (5%) for 6 negative samples passed the criterion. Averaged hydrophobic values and the distance of hydrophobic pattern for 199 aligned regions are shown in Fig. S1b. We noticed that hydrophobicity criterion can discriminate alignments of negative samples in the moderate range of hydrophobicity (0~0.5), in which the averaged hydrophobic values of KDTs are localized. These results demonstrate how the two step criteria works well.

Table S5 revealed limitations of the RW predictor that requires both of α helices and β strands for positive predictions. In principle, it is difficult to detect obligate substrates of all α or all β proteins. Actually, the structure of dTDP-4-dehydrorhamnose 3, 5-epimerase (JW2023) comprises mainly β strands and the structure of uncharacterized protein YfdR (JW2358) is mainly composed of α helices. In both cases, no alignment could not pass the structure criterion. We suppose that containing suitably arranged α helices and β strands with appropriate hydrophobic pattern in the entire structure, similar to KDTs, is one of the significant features to discriminate the obligate substrates from others, but it is not all. Other features should be explored.

Performance of predictors at various parameter sets

The list of predictors and their MCCs measured at the specified parameter set are presented in Table S2. Only the notable parameter sets, in particular those performing rather better, or the reference methods are enumerated. The results of the best-hit method are summarized in Table S3.

The RW+RR predictor

KDTs: Each KDT used in the RW+RR predictor is summarized in Table S6. The predictor is the unit of 5 KDTs: two of them (KDTs 5, 9) are derived from the structure comparison with the RW mode, and three with the RR mode. Three KDTs were selected by the structure comparisons of the TIM β/α barrel fold (c.1) and others. The backbones of 4 KDTs were substructures from either the TIM β/α barrel fold (2 KDTs) or the PLP-dependent transferase-like fold (2 KDTs, c.67). The structures of the KDTs are illustrated

in Fig. S2, where the MICAN RR mode was applied in the structure superimposition (top panel).

Relationship with RW predictor: To clarify the role of each KDT, we prepared a 5-circle Venn diagram (Fig. S3a) indicating how KDTs detect positive samples. It revealed that KDTs 5 and 6 mainly detect substrates adopting the TIM β/α barrel fold, but they are not exclusive like KDT1 in the RW predictor (Fig. 2c), which only detects structures in the TIM β/α barrel fold. Except for KDT9, KDTs detect multiple samples adopting the TIM β/α barrel fold. To investigate the roles in more detail, we compared the KDTs in both the RW and RW+RR predictors. The KDT1-9 columns in Table S1 are the structure-KDT matching-profiles, indicating which KDT was found in a structure. We analyzed the profiles of positive samples (P in the Positive/Negative column of Table S1). We compared two profiles of KDTs corresponding to positive samples and calculated the Jaccard index, a similarity measure of two digital data for two KDTs, defined by

$$JI\ (A, B) = \frac{P(A) \cap P(B)}{P(A) \cup P(B)},$$

where P(A) and P(B) are the sets of positive samples detected by KDTA and KDTB, respectively. Using "1-JI" as the distance, we applied the average-linkage clustering, and obtained a dendrogram (Fig. S3b). The illustration indicates that KDT5 is the most similar detector to KDT1 (JI=0.677). We concluded that the main TIM detector is KDT5, and KDT6 is its supporter. Like KDT3, KDT7 only identifies samples in α/β proteins. KDTs 8 and 9 act as predictors for outliers. Since the specialization of each KDT in the RW+RR predictor is schematically similar to that of the RW predictor, this scheme is likely to be universal for the development of an excellently performing predictor of GroE obligatory substrates. It also implies that the GroE obligatory substrates are structurally classified into the TIM β/α barrel fold, α/β proteins, and others.

Correlation between positive prediction rate and ΔSol: Fig. S4 shows the positive prediction rate of the RW+RR predictor against ΔSol, in the same manner as in Fig. 3 (RW predictor). For the samples of ΔSol > 30 %, the correlation coefficient is 0.84 when data with small denominators (less than 10) are removed.

Aligned regions in TIM β/α barrels: KDTs 5 and 6 are the main and the support templates to identify GroE obligate substrates adopting the TIM α/β barrel fold. For these KDTs, hit regions against TIM α/β barrel folds were examined (Fig. S7). Both KDTs tend to hit the middle part of TIM barrels, corresponding to the folding core experimentally identified. The plot is schematically similar to Fig. 6 that was illustrated for KDTs 1 and 2 in the RW predictor.

Alignment of newly identified samples and KDTs

"New fold" in GroE substrates: RumB and BioC were the first identified GroE obligatory substrates adopting an S-adenosyl-L-methionine-dependent methyltransferase fold (c.66, Fig. 5a). RumB and BioC were respectively identified by the RW and RW+RR predictors. Specifically, KDTs 3 and 4 selected RumB, and KDT7 identified BioC (Table 1). The alignment regions of RumB-KDT3 and RumB-KDT4 were adjacent SSEs in the RumB sequence and did not overlap (Fig. S5). Interestingly, the alignment region of BioC-KDT7 was their intermediate, sharing 1α1β of the RumB-KDT3 alignment, and 2β of the RumB-KDT4 alignment. This observation indicates that KDTs detect very localized regions within the whole fold, implicitly indicating the schematic similarity of KDTs. The similarity of the alignment was also observed in NrdE (c.7: PFL-like glycyl radical enzymes fold, Fig. 5b) and IdnR (c.93: Periplasmic binding protein-like I fold, Fig. 5c). KDTs 6 and 7 in the RW+RR predictor detected the NrdE structure, and the two alignments share 2β. KDTs 5 and 8 detected the IdnR structure, and the alignments share 1α1β. The remaining one is UbiX (c.34: Halotolerance protein Hal3, Fig. 5d), identified by only a single KDT.

TIM β/α barrel fold: Three proteins adopting the TIM β/α barrel fold were detected by the prediction. While BglB was evaluated to be a GroE-dependent substrate, AscB and YcdM were not (Table 1). In the former case, KDTs 1 and 2 in the RW predictor detect the protein, and both alignments highlighted the middle part of the sequence, corresponding to the folding core region of the TIM β/α barrel fold. In AscB, KDTs 1 and 3 in the RW predictor, and KDT5 in the RW+RR predictor detected the proteins. The first two KDTs selected the mixture regions around the N and C termini, and the last KDT picked the C terminal region. In YcdM, KDT5 matched regions around the N and C termini. The middle regions of AscB and YcdM did not fit any of the KDTs, implying

that the folding core structure is dissimilar to that of the positive TIM β/α barrels. We noticed that in some structural alignments of AscB and YcdM, the KDTs selected the folding core. However, the regions cannot satisfy the criterion of the KD hydrophobicity pattern.

a

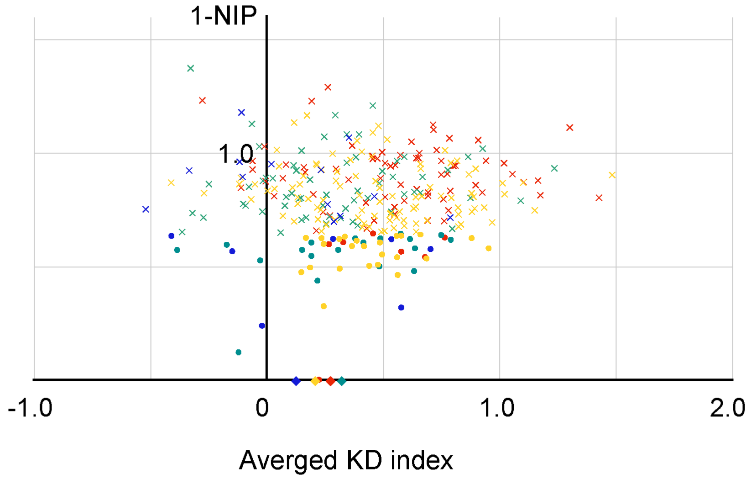

b

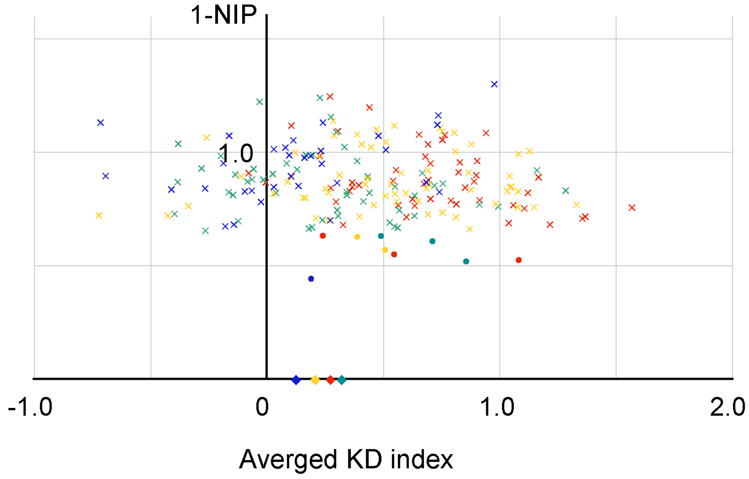

Fig. S1: Results of hydrophobic criterion. The match of hydrophobic pattern (1-NIP) and the averaged KD index for the aligned regions are plotted. (a) Positive samples. (b) Negative samples. Circles and crosses are the plots of alignments that passed the hydrophobic criterion and that did not, respectively. Plot of alignments against KDT1, 2, 3 and 4 are colored by orange, green, red and blue, respectively. Diamonds are the plots for KDTs, colored with the same manner to the alignments.

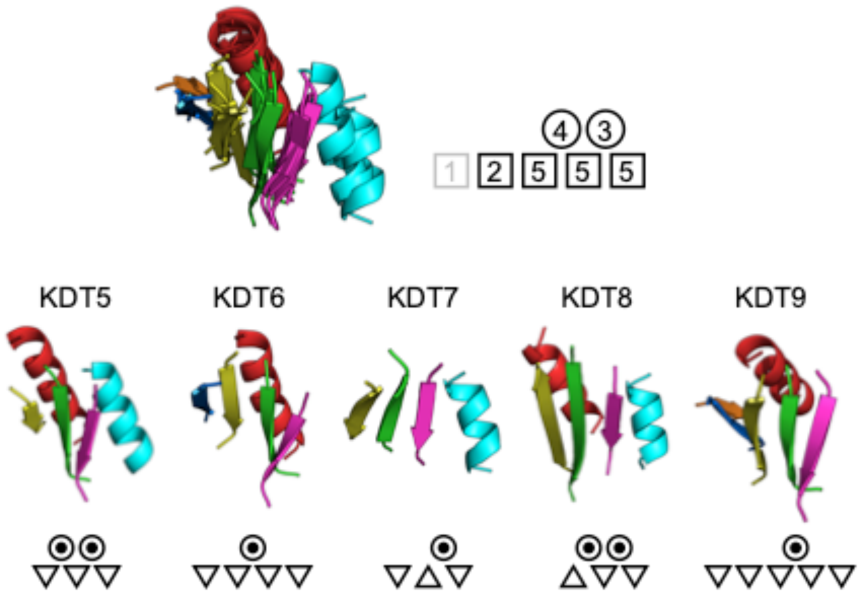

Fig. S2: KDTs used in the RW+RR predictor.

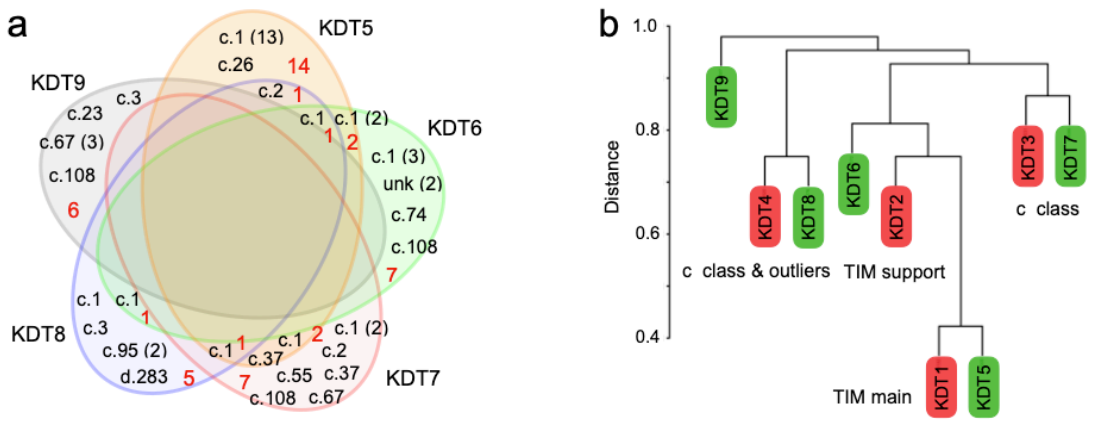

Fig. S3: Role of KDTs used in the RW+RR predictor. (a) Five-circle Venn diagram showing how many positive samples (labeled by SCCS) are detected by each KDT used in the RW+RR predictor. SCCSs are shown in black characters. Multiple hits are denoted in parentheses. Red numbers are the number of positives in each box. (b) Similarity of the roles of KDTs. The similarity of two KDTs was estimated by the preferences in the positive-sample detection using the Jaccard index. The roles of KDTs are divided into: the main TIM (c.1) predictor, the supportive TIM predictor, the α/β protein predictor, and the outlier predictor.

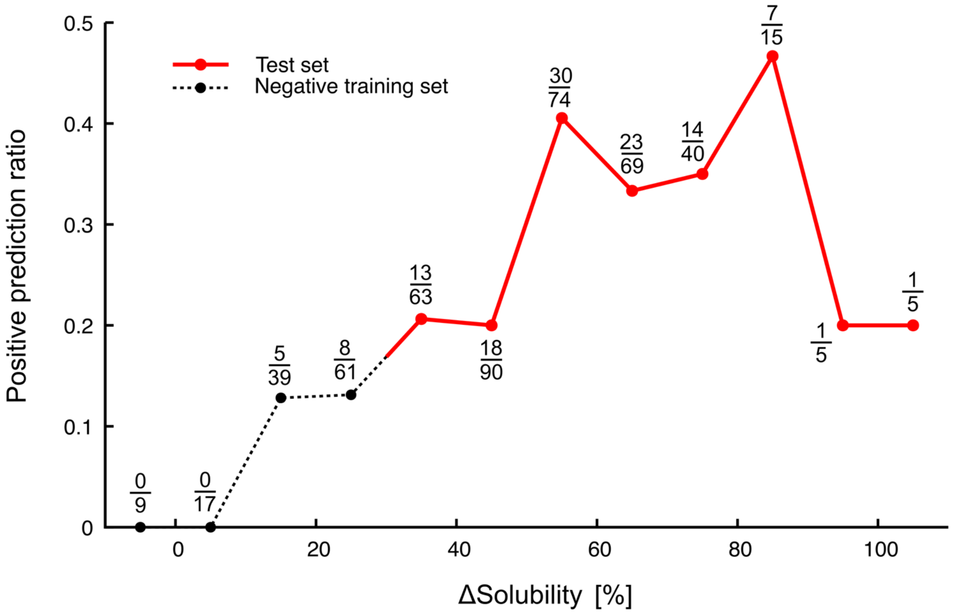

Fig. S4: Positive prediction rate by the RW+RR predictor against ΔSol.

```
KDT7        ..............................HHHHHHHH...............................................
SSE(BioC)   ........HHHH.........EEEEE.....HHHHHHHHH..EEEEEE..HHHHHHHHHH........EEEEE............
BioC(29)    .....QSADALLAMLPQRKYTHVLDAGCGPGWMSRHWRERHAQVTALDLSPPMLVQARQKD.....AADHYLAGDIESLPLATAT
RumB(215)   PAVASQLYATARDWVRQLPVKHMWDLFCGVGGFGLHCATPDMQLTGIEIASEAIACAKQSAAELGLTRLQFQALDSTQFATAQGD
SSE(RumB)   HHHHHHHHHHHHHH.......EEEEE.....HHHHHHH....EEEEEE..HHHHHHHHHHHHHH....EEEEE............
KDT3        ...HHHHHHHHHHHHH.....EEEEEEEHHHHHHHHH.......EEEEEE....................EEEEE..........
KDT4        .....................................................................................

KDT7        ..EEEEEE....................EEEEEE....................................EEEEEE
SSE(BioC)   E.EEEEEE......HHHHHHHHHHHEEEEEEEEE.....HHHHHHHHH...EE...EE.........EEEEEEE..
BioC(104)   F.DLAWSNAVQWCNLSTALRELYRVVRPVVAFTTLVQGSPDEIEQSLNGVHHHIQPITLWFDDLTYHLFLGVIARE (251)
RumB(300)   VPELVLVNPPRRGIGKPLCDYLSTMAPRFIIYSSCNAQTMAKDIRELPGFRIERVQLFDMFPHTAHYEVLTLLVKQ (375)
SSE(RumB)   ...EEEE........HHHHHHHHHH...EEEEEE..HHHHHHHHHH....EEEEEEEE.........EEEEEEE..
KDT3        ..EEEEEE....................................................................
KDT4        ...................HHHHHHH.EEEEEEEEE.HHHHHHHHHHH.EEEEEEEEEEE....EEEEEEEEEEE.
```

Fig. S5: Hit regions between RumB, BioC and KDTs. RumB and BioC adopt the same fold (c. 66), and they are alignable. MICAN aligned both proteins. KDTs 3 and 4 hit RumB, and KDT7 hit BioC. Note that KDTs are only composed of SSEs. α helices and β strands are colored orange and green, respectively.

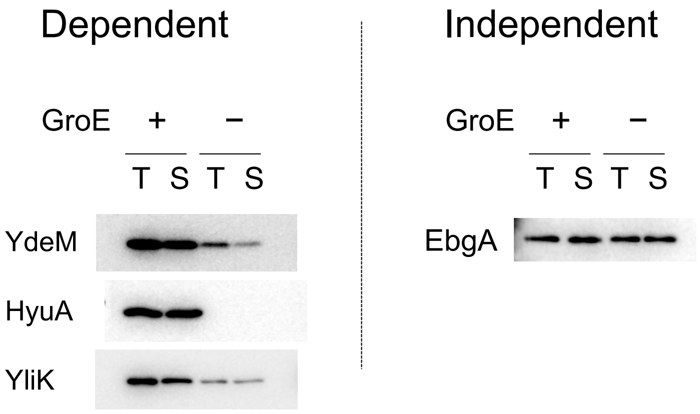

Fig. S6: Evaluation of GroE dependencies *in vivo* for four proteins adopting TIM β/α barrel fold in the negative samples (JW1492; YdeM, JW2841; HyuA, JW2884; YliK, JW5511). Each protein was expressed under GroE+ and GroE- conditions and analyzed by a centrifugation-based method (see Materials and Methods). "T" and "S" represent the total and supernatant fraction, respectively. The proteins with decreased intensities in S of GroE- as compared to T of GroE- or decreased intensities in T of GroE- as compared to T of GroE+ were determined to be GroE obligate substrates *in vivo*.

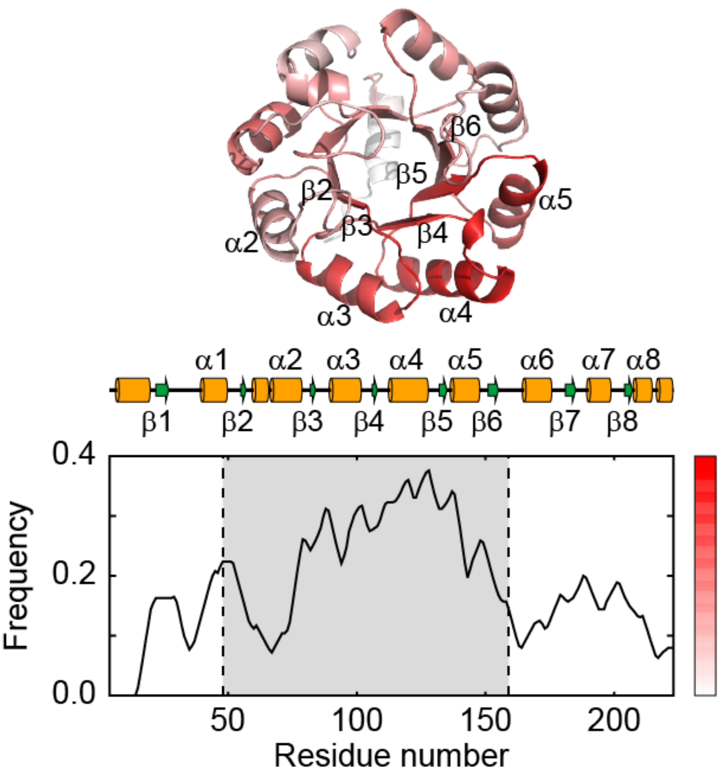

Fig. S7: Match regions of substrates adopting the TIM β/α barrel fold with KDTs 5 and 6. KDT5 and KDT6 identified 18 and 7 TIM β/α barrels, respectively. Each structure was aligned to the reference structure match of each residue against KDTs was counted (see the legend of Fig. 6). The folding core of TIM β/α barrels is highlighted in gray. In the upper panel, the reference structure is illustrated, in which the higher match frequency is colored red (the right bar).
